# In Silico Modeling of the Immune System: Cellular and Molecular Scale Approaches

**DOI:** 10.1155/2014/371809

**Published:** 2014-04-06

**Authors:** Mariagrazia Belfiore, Marzio Pennisi, Giuseppina Aricò, Simone Ronsisvalle, Francesco Pappalardo

**Affiliations:** ^1^Department of Drug Sciences, University of Catania, V.le A. Doria 6, 95125 Catania, Italy; ^2^Department of Mathematics and Computer Science, University of Catania, V.le A. Doria 6, 95125 Catania, Italy

## Abstract

The revolutions in biotechnology and information technology have produced clinical data, which complement biological data. These data enable detailed descriptions of various healthy and diseased states and responses to therapies. For the investigation of the physiology and pathology of the immune responses, computer and mathematical models have been used in the last decades, enabling the representation of biological processes. In this modeling effort, a major issue is represented by the communication between models that work at cellular and molecular level, that is, multiscale representation. Here we sketch some attempts to model immune system dynamics at both levels.

## 1. Introduction

The immune system aims to protect the body from infective illnesses and against the microbes (virus, bacteria, and parasites) that are recognized as extraneous. The defense mechanism of the host connects the answers given by the innate (or natural) and adaptive immune system, where cells and molecules perform together. An initial phase of defense against microbes is furnished by physical barriers, soluble mediators, and specialized killer cells. The identification of the same structures in groups of microbes is given by cellular receptors which are called “pattern-recognition receptors”; Toll-like receptors (recognize bacterial peptides, flagellin, lipopolysaccharide, and other biological elements), mannose receptors (bind the carbohydrate fragments with the pathogen), and seven-transmembrane spanning receptors (initiated by bacterial peptides or by endogenous chemokines) are the elements of this composition of cellular receptors.* Epithelial surfaces* (skin and mucosal surface of the gastrointestinal and respiratory tracts) and the* blood coagulation systems* are the physical barriers that are utilized as filters for extraneous agents. In the innate immune system the specialized effector cells, granulocytes and macrophages, move towards the microbes killing them with phagocytosis. Thanks to natural killer—NK—cells these microbes can be eliminated by releasing lytic substances. The neutrophils and basophils have aimed to eliminate bacteria and parasites. The NK cells kill infected cells both by the releasing of perforins and granzymes and activating the macrophages. These elements are the protagonists of the innate immune system.

The second response which defends the organism against the possibility of becoming infected has been represented by* adaptive immunity* which is able to recognize the antigens characteristics. This is important for the successive and repeated expositions. The protagonists of this immune response are the lymphocytes. The lymphocytes family is diversified by its types of antigen receptors in lymphocytes T and B. The immunoglobulins (Ig) are glycoproteins produced by lymphocytes B and have the function of membrane antigen receptors of the B cells. The antibodies are connected to the antigens binding a complex insoluble antibody-antigen, readily removed from the blood. Besides these functions, the antibodies have the capacity to also bind the cellular membrane to activate the complementary components, taking them to the cell lysis. TCR is the surface antigen receptor of T cells. TCR recognizes the peptides presented on the cell membrane of the major histocompatibility complex (MHC) and subsequently it activates a signaling cascade. The cytotoxic T cells (Tc, CTL, CD8+) kill cells expressing the antigen, the helper T cells (Th, CD4+) regulate the activity of B and Tc cells, and the regulatory T cells inhibit immune response; these represent the subpopulations of T cell. It is important to remember that the helper T cells which are subdivided in Th1 cells release the *γ*-INF and cytokines, while Th2 cells release interleukin 4 (IL-4); they stimulate the immune response against viruses, intracellular bacteria, and parasites. Antigen presenting cells (APC) capture and process the foreign proteins, and afterward they present them at Th cells with MHC molecules. This event evolves in the activation of Th cells that proliferate and secrete cytokines. These cytokines activate the antibody production by B cells and replication of Tc cells. All these elements reach the periphery where they bind and kill the pathogens and infected cells. When a lymphocyte recognizes an antigen, it leaves a population of memory cells (primary immune response) behind; in the second phase these memory cells respond rapidly to subsequent encounters (secondary immune response) [1].

Processing and analysis of biotechnology and information technology data are accelerating the expansion of knowledge of biological systems. These data are changing the way biomedical development, research, and applications are done. The set of clinical and basic biology data is important for the descriptions and modeling of disease progression, response to therapies, and in general for the diseased states. Biomedical data produced by high-throughput genomics, proteomics, and immunomics projects are analyzed and studied thanks to computational models. These computational data are important for the simulation of biological processes and these amounts of data enable the study of various levels of biological organization, from cells to the whole organism. The mathematical modeling, in biology framework, is used to analyze and to generate verifiable predictions, test hypotheses, and virtual experiments. For example, the same techniques were born in the last years, like virtual screening, docking as far as evolution of dynamic simulations, steered molecular dynamics (SMD) [2], or metadynamic studies.

To obtain a description of the different mechanisms governing the behavior and causality relations among the various parts of a living system, mathematical models at different space and time scales are required [3]. The first step for designing the mathematical model of a natural phenomenon is to define the mathematical variables. The principle lex parsimoniae, or most commonly known as the* Ockam's Razor*, is used to describe a phenomenon. For an exact analytical approach we use the classical mathematical models, but besides these, another modus operandi is now commonly employed, which we can call the* synthetic approach.* This approach is not new, because Leonardo Da Vinci used this to construct toy models of flight machines, before attempting a real scale. This* synthetic approach *consists in constructing a* replica or toy *of the studied system in terms of the laws that govern the relationship among the elements of the system. This “digital synthetic” approach is defined as simulation, for the use of digital computers, which are extremely powerful to execute difficult algorithms, these representing the entities, laws, and all kinds of experiments that can be made. Well, if the complexity of the initial toy model is augmented by simply adding new entities and laws, the Ockam's principle is still valid and used in the first phase of the studies of a certain natural phenomena. We can arrange models by describing reality at different scales of observation, thus constructing a multiscale model. The concept of multiscale models is fundamental for understanding human physiology in disease conditions. The choice of the observation scale is an important step in science. When “measuring” nature, we choose a temporal and a spatial scale to make a valid observation. In biological phenomena the complexity arises from the fact that changes at lower scales modify the way in which those elements will play at higher scales. In the study of complex phenomena we can understand why the human pathologies are very complex, for the various mechanisms in action and for the casual relationships among different in the components immune system. The models that try to describe these systems usually fix the spatial and temporal scale and use the mathematical or computational formalism. However, the computers provide the dynamics by executing the rules described in the mathematical formalism. The problem is that the real system is not isolated; indeed the alteration of only one element reverberates on the whole system. The immune system can be observed different scales; this allows us to understand how the lower levels affect the higher levels. For example how the effect of specific molecule goes to influence the behavior of a cell. A model can be formulated by using two main approaches, top-down and bottom-up.

The bottom-up approach is adaptive, robust and is suitable for studying the interaction of elements in the systems. This approach is excellent when we choose to take into account the individual elements and their interactions in the biological system.

The top-down approach considers not only the details of the individual elements, but it shows the system at the macroscopic level. During the formulation of the model, it uses experimental observations as guidelines. The advantage of this approach is the simplicity, but it sometimes happens that it does not correctly reveal the actual responsible mechanism.

An alternative to differential equations can be represented by Gillespie's algorithm. These algorithms simulate with a good chemical or biochemical system of reactions, generating statistically correct trajectories. The tissues and the whole organs are modeled in two ways: as functional compartments of system units, or as a collection of microscopic components.

In the first case the organ is represented as a black box, for which the relation for each input and output is known. This relation is developed by differential equations. Briefly the term tissue is intended as an array of individual units, which exchanges signals with the environment. This multicellular system has been developed to study the growth of solid tumor. Another example of this system is in the framework of the hemodynamic; we can use the Navier-Stokes equations for the postoperative hemodynamics issues in congenital heart diseases.

The ultimate task of multiscale modeling is not just about developing models at different scales but to link them in a consistent manner, so that the information from a lower scale can be carried into the simplified model at a higher scale.

Ordinary differential equations are used for describing the time evolution, partial differential equations are concerned with the evolution of systems, and kinetic theory approach is concerned with the modeling of interactions among the entities; these are the models used in the multiscale system. However a completely multiscale description is reached only if these models are linked together. The asymptotic method is an alternative approach for deriving macroscopic equations. In the development of asymptotic methods, the first step is the choice of the time-space scaling. Afterwards the distribution function is expanded in terms of a small dimensionless parameter. Finally the asymptotic limit is performed. The kinetic equations lead to the macroscopic system, drift—diffusion, where the diffusion processes dominate the behavior of the solutions. Finally the use of kinetic models coupled with deterministic thermostats has recently been proposed for the modeling of complex biological systems [4].

To help interpret the biomedical data produced by high-throughput genomics and proteomics projects, the scientists used the mathematical and computational models. Thanks to the advance of computer models, the simulation of complex biological processes has been achieved. In this context “system” and “model” are the keywords.

A system is a term that defines a collection of interrelated objects; this term in biology is a collection of different cells that are specialized for a specific biological function.

A model is a description of the relationship among the objects, which form the system. A system is an unknown “black box,” which under a specific stimulus (input) produces a response (output). We focus on the mathematical representation of the system. The models for technical use are formal models; these types of models are called the black box models (BBM).

Actually, one can identify four different types of models. The* verbal models* are used in the first approach to analyze the biological system. A verbal model underlines in a simple way the objects and relations among them in the system. In the* conceptual* or* diagrammatic models* (CM) the system is described by a graphical representation of the objects that describe the dynamical processes. Unified Modeling Language (UML) and Object Modeling Technique (OMT) can be used for describing a conceptual model. When we use UML the conceptual model is often described with a class diagram in which classes represent concepts and the role-type of an association represents role types taken by instances of the modeled concepts in various situations.

The* physical models* are used when the properties of the system are almost “scale-invariant.”

In the* formal models *the mathematical representation of the model depends on the knowledge of the system, on modeling choices, and the aim of the modeling process. The model may or may not consider the evolution of the system with respect to time.

A system is viewed as an assembly of different parts or compartments with different functions; these are called “compartment models,” which may pick a different mathematical representation.

The task of finding a good model is an issue. Understand the problem, devise a plan for solving the problem, execute the plan, check the correctness of the answer, and eventually refine the model representing the four principal steps for the modeling process. In these steps, it is necessary to include the model implementation, the use of the model to forecast the system, and the model adherence to reality by matching predictions with available data. The natural phenomena can be observed at different scales; indeed in describing the phenomenon by conceptual and quantitative models one needs to choose an appropriate scale.

A basic unit of a scale is the “cell,” without its physical dimension. Thanks to this concept, one can define different scales, the “cellular scale,” the “subcellular scale,” the “mesoscopic scale,” the “populations scale,” and so forth. Modeling the activity of a single cell is a very hard problem. In these cellular scales we focus on the description of the evolution of a system and in the cell interactions, which are regulated by signals emitted and received by cells. One of the requirements to be considered to model a system is a network of organs that perform a specific task. A model of a biological system can be arranged on different levels. The effects of all scales and the effects of the environment are reflected on a single organism, which can modify the overall dynamic of the populations. A collection of different parts forms a living organism organized in a complex system. Indeed both the whole organism and also parts of it are too complex to be represented in a single, precise multiscale model. Thus it is only advisable to break the conceptual model into a set of small models that describe only a part of the phenomenon, and after that connecting their outcomes [5].

## 2. Examples at Cellular Level

The revolutions in biotechnology and information technology have produced clinical data, which complement biological data. These data enable detailed descriptions of various healthy and diseased states and responses to therapies.

For the investigation of the physiology and pathology of the immune responses, computer and mathematical models have been used in the last decades, enabling the representation of biological processes.

Computational models are relevant for the understanding of biological systems. Such models can be used to optimize or predict therapeutic effects at the organism level. The pharmaceutical companies suggest that computational biology can play an excellent role in this field. To the complexity of the immune system, one needs to use the silico models that can provide answers to the general behavior of the immune system, the analysis of cellular and molecular interactions, the effects of treatments, and the course of diseases.

The use of mathematical/computational models has been requested both to perform in silico experiments which lead to formulate and/or validate biological hypotheses and to give useful hints for the design of optimal treatment schedules. Among these modeling techniques, agent based modeling (ABM) represents a bottom-up approach that can be used at cellular level to describe complex systems in a flexible way.

The agent based modeling approaches handle entity heterogeneity and physical space. These modeling techniques try to recreate and predict the cellular interactions simulating the behavior and the interactions of autonomous entities (cells and molecules). The dynamic agents can be described as a function of time, a position, and an internal state that includes most important properties of the agent, such as age. Thanks to ABM, it is easy to describe complex behaviors, but they usually lack a solid mathematical basis and require huge computational resources in order to allow natural scale simulations. The success of the ABM has allowed its use for simulating many pathologies such as HIV [6], tumors [7–9], atherosclerosis [10], and multiple sclerosis [11].

As a first example we will focus on an agent based model that works at cellular scale. This model shows how the regulatory T cells (Treg) and effector T cells (Teff) cross-regulation mechanism is essential in the understanding of the evolution of an appalling disease, multiple sclerosis.

Multiple sclerosis (MS) is a disease of the central nervous system. This disease causes a reduced communication among nerve cells, causing the removal of the myelin sheath from axons of the brain and spinal cord. There are different causes that play an important role in this disease. Autoimmunity is usually considered the principal actor, since its attacks on the myelin sheath cause the damage or genetic factors including HLA-DR15, HLA-A∗02, and HLA-DRB1∗1501 and have a significant role. Furthermore Epstein-Barr viral infection, some dietary factors, pharmaceutical therapy (immunotherapy), and behavioral factors (stress) play a negative role in the disease. A protective role in MS and neurodegeneration is given by vitamin D and turmeric. These factors are important to prevent MS, because the turmeric and vitamin D protect the brain from destruction of the myelin sheath.

The disease occurs mostly in women (from the age of 20–40). Vision problems, fatigue, dizziness, difficulty in muscle coordination and losing strength, uncontrolled bodily functions, and weakness of limbs are the symptoms of the disease and they cannot easily be predicted. It is very hard to understand the response to treatment. Around 90% of all the patients have relapsing remitting multiple sclerosis (RRMS). It is the prevalent type of MS, where relapse and remission occur after a certain time period. Relapse is defined like a period of worsening of disease activity with the development of new symptoms or reoccurrence of previous symptoms. The partial or complete recovery of the symptoms following relapse is defined as the period called remission. It is showed that, in the progressive phase of the disease, 80% of cases are followed by chronic disease progression. Expanded Disability Status Score (EDSS), magnetic resonance imaging (MRI) lesion, and other physical tests including timed 25-Foot Walk and MS Walking Scale-12 are studies that show the progressive phase of the disease.

In disease progression the T cells play a principal role; this information is given by the documentation that regulatory T cells decrease in the blood when relapse occurs, while the helper T cells increase in spinal fluid. Regulatory T cells and effectors T cells play an important role in preventing autoimmunity.

Deficiency or lack of functionality of Treg may cause negative effects in the peripheral tolerance mechanisms; these control the activation and proliferation of Teff.

The ABM model that describes the typical spikes observed into RRMS [11] works under the hypothesis that the cross regulation between Teff and Treg represents a crucial point in the appearance of the disease. This process causes an oscillatory behavior in healthy patients, which leads to unrecoverable neural damage in patients with a malfunction in the cross-regulation mechanisms.

In particular, it has been supposed that Teff cells are downregulated by Treg cells and Treg cells are upregulated by Teff cells. This cross regulation reminds us of the predator-prey (Lotka-Volterra) equations. The preys are represented by activated Teff cells that use myelin and cerebral tissues for growth, whereas predators are Treg cells that try to catch Teff cells.

Beyond the presence of a mechanism of competition and cross regulation between Treg and Teff, some other experimental hypotheses have been made.

Among these, the presence of a virus that causes an inflammation represents another biological hypothesis. The EBV is a perfect candidate; it has been identified to be responsible for MS, perhaps that EBV-specific T cells could cross-react with autoantigens expressed in the SNC and attack the myelin sheath of axons.

The second example we will provide refers to a model of the immune response elicited by a cancer vaccine against tumor.

The immune system includes many cells and molecules that cooperatively act to protect the host organism from foreign agents. Tumors are dynamic complex systems in which several entities, events, and conditions interact among them resulting in growth, invasion, and metastases.

The immune system can eliminate most of the cancer cells; however, those cells that are not recognized and escape immune surveillance represent the target of all anticancer treatments. The ultimate goal of tumor vaccines is to ultimately stimulate an immune response against poorly immunogenic tumor variants.

Vaccines for cancer differ from the traditional vaccines, which instruct the immune system on how to recognize and destroy a particular pathogen. Cancers vaccines indeed enlist the patient's immune system to destroy existing cancer cells.

Cancer vaccine aim is usually to activate a component of the immune system such as B lymphocytes, which produce anticancer antibodies, or T lymphocytes that directly kill tumor cells. The antibodies recognize antigens and once bound are capable of destroying tumor cells by means of antibody-dependent cellular cytotoxicity or complement-mediated cytotoxicity.

Fighting cancer is possible only by understanding the interactions between a tumor and the immune system. Actually, a well-known approach in cancer immunotherapy is the use of cytotoxic T cells (CTLs), dendritic cells (DC), and antibodies. The approach with the anti-idiotype (Id) antibodies as vaccines stimulates the immune system response against tumors. The studies in mouse tumor models have shown that DCs pulsed with tumor antigens can induce protective and therapeutic antitumor immunity. It is essential to mention the manipulation of dendritic cells to achieve protective or therapeutic immunity. The prophylactic vaccines have shown that their administration to transgenic mice can completely prevent tumor onset and restore a normal life expectancy. However, even if the prophylactic cancer vaccines are still far from human applications, this opens the doors to an eventual new perspective in cancer prevention.

The applications of computational simulations are important for the discovery, the design, and the optimization of vaccines. To model the behavior of a cancer vaccine, one needs to model the immune system, and this is very complicated, because it represents one of the most complex biological systems.

A considerable step in this field has been represented by the realization of the SimTriplex model. The model represents the first example in modeling a cancer vaccine. It is an agent-based model that simulates the effects of “Triplex” tumor-preventive cell vaccines in HER-2/neu transgenic mice prone to the development of mammary carcinoma. When the Triplex vaccine is administrated to BALB-neuT mice, it blocks the mammary carcinogenesis, allowing a survival of the whole organism. The limitation of the Triplex vaccine is given by the fact that the only a chronic protocol (more than 60 vaccinations) blocks the mammary carcinogenesis, while a shorter and/or delayed protocol can lead to the tumor onset in mice.

A successful vaccination schedule should be able to prevent the formation of the solid tumor in the host for the entire lifetime. The characteristics of the vaccine action are to prevent the development of a solid tumor mass and to induce an immune response that controls the number of tumor cells, particularly at the first appearance of the tumor cells. If a solid tumor is already established, Triplex vaccine is not effective.

The SimTriplex model coupled with optimization techniques [12, 13] allowed to characterize an optimal vaccination schedule. The results shown by in vivo validation of SimTriplex previsions showed that better protocols are possible and can be suggested by computational models, even if further adjustments to the model were still required.

Further studies on the Triplex vaccine have then showed that the efficacy as a therapeutic vaccine, but also as the use against induced lung metastases. A new computational ABM model, called MetastaSim [9, 14], has been then developed and used as an in silico virtual lab. The suggestions that arose from the use of MetastaSim showed that, in order to prevent lung metastases formation, optimal administration protocols should be composed by an initial massive vaccine dosage followed by few vaccine recalls. Even if such a vaccination strategy, which is composed by three vaccine injections, a period of rest, and then a series of vaccine recalls, is commonly used in designing of vaccine protocols, it represented a new finding in the field of cancer vaccines immunotherapy, and thus it has been considered as a relevant result.

Furthermore, the possible effect of an immunotherapy based on the transforming growth factor beta, TGF-*β* (immunoregulatory protein), in combination with vaccine treatments has been investigated using mathematical and computational models working at cellular scale [15].

A final example of the use of computational models at cellular level is represented by the induction of immunity using a technique that is known as “dendritic cell transfection” [13]. This term represents the practice of cultivating “autologous dendritic cells” (extracted from the same patient) with some known tumor associated-antigen (TAA) subsequently injected back into the patient. The resulting vaccine is called dendritic cell vaccine (DCV). As a side effect of the immune response against the DCV will be the same as the recognition TAA molecule on tumor cells and will kill them.

The numerical simulations and stability analysis of an ODE model which works at a cellular scale allowed to reproduce experimental results and also permitted to use them in designing future immunotherapy experiments. From a mathematical and computational point of view, the investigation of vaccines and therapeutic approaches against cancer represent a new field of research.

## 3. Examples at Molecular Level

In the example of molecular level we report the development of the theoretical basis and the properties of a novel predictor, NGlycPred, which predicts the glycan occupancy.

Glycosylation plays an important role in a number of biological processes ranging from protein folding to immune response. A ubiquitous co/posttranslational modification in eukaryotic cells is represented in N-linked glycosylation; this event occurs when the* nascent protein* is extruded into the endoplasmic reticulum. N-linked glycosylation occurs with the attachment of glycan to the amide nitrogen of asparagines. This process is mainly shown in the sequons N-X-T and N-X-S (X is any standard amino acid except proline). Not all N-X-T/S sequons can be glycosylated, but using an accurate algorithm could predict the glycan occupancy. However, the use of proteins structural information is important for the prediction of N-glycan occupancy. These sequons are essential for understanding and using the ubiquitous co/posttranslational protein modification.

It has been seen in the studies that, to improve the prediction of N-linked glycosylation, a number of algorithms with sequence or sequence-based techniques are essential. The random forest (RF) algorithm has been used at this end, which is based on the whole protein sequence and on the subcellular localization information. The RF algorithm is efficient and can handle numeric and nominal values simultaneously.

The glycan occupancy of N-X-T/S sequons of eukaryotic proteins is predicted in NGlycPred. RF algorithm was trained using NGlycPred on N-linked glycosylated proteins. NGlycPred is the first publicly available web server to predict N-glycan occupancy at N-X-T/S sequons.

The ability to predict N-glycan occupancy should allow for a better understanding of overall protein structure and in general of biological processes. The studies have shown that NGlycPred performed is better than the sequence-based predictors generated.

The predictors generated using structural properties are better than those generated by sequence information. This shows that the comparison between different predictors is complicated, because the predictors were developed and optimized on different datasets. Recent observations have shown that specific amino acid side chains could directly stabilize the first N-acetylglucosamine of the glycan. The sequons were only selected from PDB files where the proteins were expressed in eukaryotic systems, because the eukaryotic and prokaryotic N-linked glycosylation schemes are different. Considering glycosylating the sequons without ASN-NAG linkage from the PDB files, the incidences of false negatives were reduced. However, when the glycosylation site was occupied and the glycan electron densities were absent, the false negative sequons could still be present in the dataset. The knowledge of side chain torsion angles improves the prediction of N-linked glycan occupancy, but side chain torsions are more difficult to predict. We chose not to include this feature in the models so that the NGlycPred algorithm would be applicable to both crystal structures and homology models [16].

We conclude the example of molecular level with the important function of the laminin in basement membrane. Laminins are heterotrimeric glycoproteins composed of three different gene products of *α*, *β*, and *γ* chains, with many essential functions for the membrane assembly. The adhesion to laminins is mediated by five laminin G-like (LG) domains at the C terminus of the *α* chain. The genetic studies have demonstrated their essential role in embryo development and their involvement in severe human disease, resulting from mutations in laminin genes. The three short arms are composed of one chain each; the long arm is a coiled coil of all three chains, and these contributed to form to the laminin: cross-shaped molecule. For full integrin binding to the laminin LG1-3 region, the coiled coil region and an intact *γ* chain tail are required. The studies have shown that the coiled coil region, a glutamic acid in the third position from the C terminus of the *γ* chain, and the LG1-3 region of laminin *α* chains are necessary for integrin binding. We have determined the crystal structure of laminin *α*-2LG1-3. Electron micrographs of the distal end of the long arm of the cross-shaped laminin molecules reveal LG1-3 as three globules in a triangular arrangement and in close contact with the rod-shaped coiled coil. The surprise has been that *α*2LG1-3 structure has only one such interface, between LG2 and LG3, whereas LG1 is completely dissociated from the LG2-3 pair. However, probably the result of the absence of the coiled coil and *γ* chain tail in the crystallized construct leads to this open conformation like a crystal artifact. When the peptides spanning, the *γ*1 or *γ*2 chain tails neither bind integrins nor inhibit integrin binding to laminins. This has led to electron microscopy analysis, which shows a closed LG1-3 conformation when the *γ*2 chain C terminus is intact, with an open conformation when the last three residues of the *γ*2 chain are deleted. The critical glutamic acid plays an indirect role in maintaining an active LG1-3 conformation, and it is not directly ligated by integrins. Most of the studies were carried out for understanding the integrin-laminin binding. The task of the analysis was that the integrin binding site should be free of obstructing carbohydrate modifications in all five laminin *α* chains. The analysis of N-linked glycosylation sites in the LG1-3 region shown like LG1 is a plausible integrin binding domain [12].

## 4. Conclusions

In this paper we have briefly described the problems faced when one wants to link mathematical or computational models across different time and length scales. The immune system is a complex biological system and this complexity arises from collective behavior and the emerging of properties at multiple levels. This initially requires the analysis of large quantities of clinical and basic biology data either acquired by direct measurements or by accessing a variety of sources. These data need to be integrated into various network models or multiscale models. Models are a fundamental step in the scientific discovery, but building a good model is a hard task.

In this work we presented some of the approaches to immune system modeling together with examples at cellular and molecular scale. For the cellular level we showed the first simple model of MS which uses the agent based modeling approach. It allows to see how the role of regulatory T cells opposed to effectors T cells and their internal dynamics may be crucial in the understanding the relapsing remitting MS. We have discussed the importance of the traditional vaccines and of cancer vaccines, in terms of dosage and timings, focusing on how the search of an optimal vaccination protocol requires the construction of mathematical and computational models of the immune response.

For the molecular level, we initially reported the development of the theoretical basis and the properties of a novel predictor, NGlycPred, which predicts the glycan occupancy. The ability to predict N-glycan occupancy should allow for a better understanding of overall protein structure as well as the biological processes and may also assist in the design of hyperglycosylated immunogens. In the final example—the laminins, which are large heterotrimeric glycoproteins with many essential functions in basement membrane assembly and function, we showed how their essential roles in embryo development and tissue function have been demonstrated by numerous genetic studies and the analysis of severe human diseases resulting from mutations in laminin genes.

We would like to conclude by stressing the interdisciplinary nature of the experiences described above and by noting that the contribution of life scientists needs to go beyond the data supply. It is extremely important to take into consideration the biological scenario and mathematical or computational data to construct robust and valid models.
